# Hipertensão e Risco Cardiovascular: Associação Direta com os Valores Pressóricos

**DOI:** 10.36660/abc.20240459

**Published:** 2024-09-04

**Authors:** Rui Póvoa

**Affiliations:** 1 Universidade Federal de São Paulo São Paulo SP Brasil Setor de Cardiopatia Hipertensiva da Universidade Federal de São Paulo, São Paulo, SP – Brasil

**Keywords:** Doenças Cardiovasculares, Hipertensão, Idoso

A hipertensão arterial (HA) é um dos principais fatores de risco das doenças cardiovasculares (CV), principalmente o acidente vascular cerebral, a doença arterial coronária, insuficiência cardíaca e insuficiência vascular periférica.^1^ O controle adequado dos níveis pressóricos reduz de forma significativa o aparecimento de todos estes eventos nefastos.

Em vista da pressão arterial (PA) ser uma variável biológica contínua a definição de normalidade ainda não é um consenso universal. A última diretriz brasileira de HA publicada em 2020 considera hipertensos os indivíduos com PA ≥140/90 mmHg (sistólica/diastólica).^2^ Entretanto sabemos que o risco CV começa a se elevar a partir de valores pressóricos bem inferiores aos considerados como normalidade.

Os autores da diretriz da “American College of Cardiology/American Heart Association” (ACC/AHA) que foi publicada em 2017, preocupados com o risco CV crescente com os níveis pressóricos, fizeram uma nova classificação do hipertenso. Consideram a PA normal se <120 mmHg (sistólica) e <80 mmHg (diastólica), PA elevada entre 120-129 (sistólica) e <80 mmHg (diastólica) e hipertensão estágio 1 valores entre 130-139 (sistólica) ou 80-89 mmHg (diastólica) e estágio 2 PA≥ 140 mmHg (sistólica) ou 90 mmHg (diastólica).^3^ São valores que se contrapõem a diretriz brasileira. O estágio 1 da ACC/AHA praticamente é similar ao que consideramos como pré-HA e pela nossa diretriz se este paciente for de baixo risco não há indicação formal de tratamento farmacológico.

O trabalho de Lu et al. realizado na população chinesa, verificou se a classificação da ACC/AHA, acrescentava alguma informação sobre o risco CV nos níveis pressóricos por eles considerados normais.^4^ A diretriz da “Liga Chinesa de Hipertensão” também considera HA se a PA≥140/90 mmHg.^5^Além disso, havia diversas controvérsias do risco CV na população chinesa nos diversos estágios da ACC/AHA nos indivíduos de meia idade e nos idosos. Os resultados mostraram que naqueles indivíduos classificados pela ACC/AHA, com PA elevada, estágios 1 e 2, os riscos CV eram bem mais elevados e tinham relação crescente com o nível pressórico.

A população chinesa difere da brasileira em diversos aspectos (étnicos, culturais, alimentares, religiosos, etc.), mas por outro lado apresenta alguma semelhança em relação a prevalência de doença CV e envelhecimento populacional. Desta forma resultados podem ser transpostos, com algumas ressalvas, para a nossa realidade. Evidentemente que um cuidado a mais nos pré-hipertensos (considerados hipertensos estágio 1 pela ACC/AHA) pode trazer uma maior proteção CV.^6^

A pré-HA já foi investigada no sentido do risco de o paciente evoluir para a HA declarada. Julius et al. no estudo TROPHY verificaram que o tratamento farmacológico com bloqueadores do receptor da angiotensina II durante quatro anos reduziu a incidência de HA.^7^Lüders et al., no estudo PHARAO avaliaram se o uso de inibidores da enzima de conversão da angiotensina I eram efetivos nos pré-hipertensos e encontraram resultados positivos.^8^

Nestes pré-hipertensos encontram-se uma quantidade expressiva do fenótipo HA mascarada (PA de consultório <140/90mmHg e a medida fora pela MAPA ou MRPA ≥130/80mmHg).^9^ Esta condição frequente tem um comportamento tão agressivo nos órgãos alvo quanto a HA não controlada, ou até pior devido à dificuldade de um diagnóstico preciso.^10^

Barroso et al. avaliando a prevalência de HA mascarada em pré-hipertensos com o uso da TeleMRPA encontraram este fenótipo em 11,4% dos pacientes.^11^Um número expressivo de indivíduos sob risco CV elevado que podem ficar sem tratamento por falta de diagnóstico correto.

Trabalhos de aplicabilidade populacional são importantes pois podem mudar conceitos e direcionar para uma conduta terapêutica mais objetiva e precisa. Além disso, a adoção mais rígida de valores de normalidade para o diagnóstico da normotensão pode aumentar os gastos iniciais com a saúde, mas com certeza haverá muito menos eventos CV, estando a população mais protegida.

Este estudo de Lu et al.^4^ reflete a transposição para o cotidiano clínico de que níveis pressóricos pouco elevados são significantes em uma ampla esfera de agressão aos órgãos alvo. Em vista do número elevado da população afetada com a pré-HA isto pode representar um número expressivo de indivíduos com maior risco CV.

Ações pontuais, enfocando este problema, podem trazer resultados benéficos a toda a sociedade com menor número de eventos CV. Além destes conceitos de anormalidade dos níveis pressóricos, devemos sempre lembrar da importância de atingir as metas pressóricas preconizadas no tratamento, onde teremos maior redução de eventos CV com menos efeitos adversos.
