# Supplementary material for: Hipertensão e Risco Cardiovascular: Associação Direta com os Valores Pressóricos
Source: Arq Bras Cardiol. 2024 Sep 4;121(7):e20240459. [Article in Portuguese] doi: 10.36660/abc.20240459 (PMC11495811; doi:10.36660/abc.20240459)
Supplement: Translation

# Translation: Hypertension and Cardiovascular Risk: Direct Association with Blood Pressure Values

**DOI:** 10.36660/abc.20240459i

**Authors:** Rui Póvoa

**Affiliations:** 1 Brazil Setor de Cardiopatia Hipertensiva da Universidade Federal de São Paulo, São Paulo, SP – Brazil

**Keywords:** Cardiovascular Diseases, Hypertension, Aged

Hypertension (AH) is one of the main risk factors for cardiovascular (CV) diseases, mainly stroke, coronary artery disease, heart failure, and peripheral vascular insufficiency.^1^ Adequate control of blood pressure (BP) levels significantly reduces the appearance of all these harmful events.

As BP is a continuous biological variable, the definition of normality is not yet a universal consensus. The latest Brazilian AH guideline published in 2020 considers individuals with BP ≥140/90 mmHg (systolic/diastolic) to be hypertensive.^2^ However, we know that CV risk begins to increase at BP values well below those considered normal.

The authors of the “American College of Cardiology/American Heart Association” (ACC/AHA) which was published in 2017, concerned about the increasing CV risk with BP levels, created a new classification of hypertensive patients. They consider BP normal if <120 mmHg (systolic) and <80 mmHg (diastolic), elevated BP between 120-129 (systolic) and <80 mmHg (diastolic) and stage 1 HA values between 130-139 (systolic) or 80- 89 mmHg (diastolic) and stage 2 BP≥ 140 mmHg (systolic) or 90 mmHg (diastolic).^3^ These are values that contradict the Brazilian guideline. Stage 1 of ACC/AHA is practically similar to what we consider pre-AH and according to our guidelines, if this patient is at low risk there is no formal indication of pharmacological treatment.

The work of Lu et al. carried out on the Chinese population, verified whether the ACC/AHA classification added some information about CV risk at BP levels considered normal by them.^4^ The “Chinese Hypertension League” guideline also considers HA if at BP≥140/90 mmHg.^5^ Furthermore, there were several controversies regarding CV risk in the Chinese population at different stages of ACC/AHA in middle-aged and elderly individuals. The results showed that in individuals classified by ACC/AHA, with high BP, stages 1 and 2, CV risks were much higher and had an increasing relationship with BP level.

The Chinese population differs from the Brazilian population in several aspects (ethnic, cultural, dietary, religious, etc.), but on the other hand, it presents some similarities concerning the prevalence of CV disease and population aging. In this way, results can be transposed, with some reservations, to our reality. Evidently, extra care in pre-hypertensive patients (considered stage 1 hypertensive by the ACC/AHA) can bring greater CV protection.^6^

Pre-AH has already been investigated in terms of the risk of the patient progressing to declared AH. Julius et al. in the TROPHY study found that pharmacological treatment with angiotensin II receptor blockers for four years reduced the incidence of AH.^7^ Ludes et al., in the PHARAO study, evaluated whether the use of angiotensin I converting enzyme inhibitors was effective in pre-hypertensive patients and found positive results.^8^

In these pre-hypertensive patients, there is a significant amount of the masked AH phenotype (office BP <140/90mmHg and measured outside by ABPM or HBPM ≥130/80mmHg).^9^ Uncontrolled AH, or even worse due to the difficulty of an accurate diagnosis.^10^

Barroso et al. evaluating the prevalence of masked AH in prehypertensive patients using TeleMRPA found this phenotype in 11.4% of patients.^11^ A significant number of individuals at high CV risk who may remain untreated due to lack of correct diagnosis.

Population applicability work is important as it can change concepts and direct toward a more objective and precise therapeutic approach. Furthermore, the stricter adoption of normal values for the diagnosis of normotension may increase initial healthcare costs, but there will certainly be many fewer CV events, with the population being better protected.

This study by Lu et al.^4^ reflects the transposition into everyday clinical practice that low BP levels are significant in a wide range of attacks on target organs. Because of the high number of the population affected by pre-AH, this may represent a significant number of individuals at higher CV risk.

Specific actions, focusing on this problem, can bring beneficial results to society as a whole with fewer CV events. In addition to these concepts of abnormal BP levels, we must always remember the importance of achieving the BP targets recommended in treatment, which will result in a greater reduction in CV events with fewer adverse effects.
